# Efficacy of Virtual Reality–Based Mindfulness Interventions: Systematic Review and Meta-Analysis

**DOI:** 10.2196/90003

**Published:** 2026-06-24

**Authors:** Sarah Alicia Barker, Andreas J Miles-Novelo, Albert Rizzo, Regina M Tuma

**Affiliations:** 1 Psychology Department, Fielding Graduate University, 2020 De La Vina Street, Santa Barbara, CA, 93105-3538, United States, 1 800 340 1099; 2 Department of Psychiatry and Behavioral Sciences, University of Southern California, Los Angeles, CA, United States

**Keywords:** virtual reality–based mindfulness interventions, systematic review, meta-analysis, stress, psychological, affect, mindfulness

## Abstract

**Background:**

Virtual reality–based mindfulness interventions (VRbMIs) increasingly populate studies as scalable tools for stress and emotion regulation. However, findings across psychological outcomes are heterogeneous, and methodological variation in intervention design, outcome measurement, and reporting practices limits cross-study comparability and cumulative synthesis.

**Objective:**

A PRISMA (Preferred Reporting Items for Systematic Reviews and Meta-Analyses) 2020–compliant systematic review and meta-analysis was conducted to evaluate the psychological effects of VRbMIs published between 2023 and 2025, examining methodological quality and outcome consistency across diverse study designs.

**Methods:**

A PRISMA 2020–compliant systematic search was conducted across PubMed, PsycINFO, Scopus, Semantic Scholar, and CORE from January 2023 to April 2025. Eligible studies included quantitative VRbMIs reporting psychological outcomes. We assessed risk of bias using the Mixed Methods Appraisal Tool and conducted random-effects meta-analyses using Hedges *g* where data were sufficient.

**Results:**

VRbMIs were associated with a large, statistically significant reduction in negative affect and a statistically significant increase in state mindfulness, while effects on depression, stress, and anxiety were small to moderate and nonsignificant. Trait mindfulness was described narratively rather than meta-analyzed and showed limited, inconsistent change across studies. For anxiety, we included 13 studies contributing 27 effect size estimates in the quantitative synthesis. A random-effects model indicated a small-to-moderate, nonsignificant pooled effect (*g*=0.44, 95% CI –0.08 to 0.96; *t*_26_=1.74; *P*=.09), with substantial between-study heterogeneity (*Q*_26_=381.60; *P*<.001; τ^2^=0.96). Physiological outcomes were reported across a subset of studies and generally aligned with self-reported psychological findings; however, inconsistent measurement and incomplete reporting precluded quantitative synthesis. This analysis represents 35 studies with a combined sample of approximately 1550 participants (1 study did not report sample size).

**Conclusions:**

VRbMIs demonstrate statistically significant short-term benefits for negative affect and state mindfulness, with consistently positive but nonsignificant trends toward improvement in stress, anxiety, and depression. Effects on positive affect and trait mindfulness were small and less consistent. Physiological findings were promising but limited by inconsistent reporting. These results support the use of VRbMIs as accessible tools for emotional regulation across diverse populations, while highlighting the need for larger trials, standardized outcome reporting, and more rigorous control conditions to strengthen the evidentiary foundation of this rapidly evolving field.

## Introduction

### Background

Mindfulness-based interventions (MBIs) have consistently demonstrated benefits for stress reduction, emotion regulation, and overall well-being across clinical and nonclinical populations [1-3]. Virtual reality (VR) technologies provide an immersive delivery modality for mindfulness interventions through multisensory environments that support sustained attention and embodied engagement. Virtual reality–based mindfulness interventions (VRbMIs) integrate contemplative instruction with 3D or 360° environments, allowing participants to practice focused attention and relaxation within simulated natural or therapeutic contexts [1,4,5]. Early evidence indicates that VRbMIs are associated with physiological markers of regulation, including increased heart rate variability (HRV) and alpha electroencephalography (EEG) activity, alongside improvements in stress, anxiety, and mood [4,6-8]. However, findings across psychological outcomes are heterogeneous, and methodological variation in intervention design, outcome measurement, and reporting practices limits cross-study comparability and cumulative synthesis.

### Prior Systematic Reviews and Rationale for This Study

Several systematic reviews have examined VRbMIs, though each is constrained in scope in ways that limit cumulative synthesis. Ma et al [9] conducted the first narrative systematic review of immersive VR mindfulness training, including 7 studies focused specifically on whether immersion level moderates mindfulness outcomes. While foundational, that review included no meta-analysis and predates the substantial growth in the literature from 2023 onward. Wieczorek et al [4] conducted the first systematic review and evidence mapping of VRbMIs (search to September 2022), documenting consistent psychological and physiological benefits across 22 studies, including improvements in anxiety, mindfulness, affect, and stress, but explicitly did not perform a meta-analysis due to high methodological heterogeneity across studies. Milasi et al [10] reviewed 16 randomized controlled trials in nonclinical populations only, excluding the clinical, occupational, and mixed methods designs that characterize much of the recent literature. Xie et al [11] conducted a meta-analysis of 25 studies focused specifically on mindfulness as an outcome, without synthesizing the broader psychological profile of VRbMI effects across multiple outcome domains.

No completed review has synthesized empirical studies published between 2023 and 2025, incorporated psychological outcomes across diverse study designs, or evaluated methodological quality using the Mixed Methods Appraisal Tool (MMAT). This review addresses these gaps directly. By extending the search window to April 2025 and including quantitative, quasi-experimental, and mixed methods designs, this study provides an updated and more comprehensive assessment of the VRbMI evidence base than prior reviews have offered.

### Literature Context

MBIs have demonstrated benefits for psychological well-being, including reductions in stress, anxiety, and negative affect, across clinical and nonclinical populations [1-3]. As immersive technologies have matured, VR has emerged as a delivery modality for mindfulness practices that integrates guided contemplative instruction, such as breath awareness or body-focused attention, with immersive 3D or 360° environments [4]. VRbMIs typically combine standardized mindfulness guidance with simulated natural or restorative settings, enabling controlled, multisensory delivery of practice within laboratory, clinical, and applied contexts [4]. Empirical studies have shown that brief and multisession VR mindfulness exposures can reduce self-reported stress, anxiety, and negative affect, while also producing physiological indicators associated with relaxation, including changes in heart rate (HR) and HRV [4,6-8]. However, effect magnitudes vary substantially across studies, reflecting differences in intervention duration, guidance style, population characteristics, and the measurement of outcomes. These variations complicate direct comparison across trials and limit conclusions about the consistency and durability of effects.

Wieczorek et al [4] conducted the first systematic review and evidence mapping of VRbMIs, documenting psychological benefits across 22 studies, including improvements in anxiety, mindfulness, affect, and stress—alongside smaller, less consistently reported physiological effects. Notably, the authors explicitly declined to perform meta-analytic pooling due to high methodological heterogeneity across included studies. That review identified several methodological constraints, including small sample sizes, heterogeneity in intervention formats, and limited standardization in physiological reporting [4]. Notably, the review concluded before the publication of a substantial wave of studies between 2023 and 2025. More recent investigations have expanded the evidence base through the inclusion of multisession protocols, active control conditions, and improved VR hardware fidelity, reflecting maturation in both study design and implementation [5-8,10].

Across the emerging literature, researchers have applied VRbMIs in health care, educational, and occupational settings, most commonly targeting anxiety, depression, stress, affective state, and self-reported mindfulness. Physiological end points have included HRV, electrodermal activity, EEG, and cortisol measures, intended to complement subjective outcomes [4]. Despite this breadth, methodological variability remains high. Many studies rely on single-session exposures, convenience samples, or passive control conditions, while others differ in intervention dose and sensory complexity. This diversity contributes to heterogeneity in reported effects and limits the aggregation of cross-study results.

Measurement practices further constrain comparability. Researchers most often assess psychological outcomes using symptom-focused self-report instruments such as the Depression Anxiety Stress Scales, the State-Trait Anxiety Inventory, and the Positive and Negative Affect Schedule. These measures emphasize changes in distress and affective state, reflecting dominant operationalizations used in intervention research. Physiological outcomes, by contrast, are frequently reported incompletely, with missing descriptive statistics or inconsistent baselines that preclude quantitative synthesis [4]. Greater consistency in outcome selection and reporting is therefore necessary to support cumulative evaluation of VRbMIs.

To address these limitations, this meta-analysis integrates findings from the most recent wave of empirical VRbMI research, using PRISMA (Preferred Reporting Items for Systematic Reviews and Meta-Analyses) 2020 reporting standards and random-effects procedures that are suitable for heterogeneous, small-sample literatures [12]. The review evaluates methodological quality using the MMAT [13] and quantifies between-study heterogeneity. By updating the cumulative evidence base, this analysis clarifies the strength and consistency of VRbMI effects and identifies methodological priorities for future digital mindfulness research.

## Methods

### Study Design

A systematic review and quantitative meta-analysis of empirical studies was conducted to examine VRbMIs. We followed the PRISMA 2020 guidelines [12] and applied methodological standards appropriate for small-sample random-effects modeling [12,14].

### Eligibility Criteria

We included peer-reviewed empirical studies that evaluated VRbMIs in human participants. Eligible studies implemented a mindfulness-based practice delivered through immersive VR technology (eg, head-mounted displays presenting 3D or 360° environments) and reported at least 1 psychological or physiological outcome relevant to stress, affect, or well-being. We required studies to provide sufficient quantitative data to permit the calculation of effect sizes. To ensure consistent application of inclusion criteria, a mindfulness centrality rubric was developed and applied during full-text screening to evaluate whether each study’s intervention met threshold criteria for a recognized mindfulness-based practice. The mindfulness centrality rubric is provided in Multimedia Appendix 1.

While qualitative methods offer valuable insights into the subjective and phenomenological dimensions of mindfulness practice, including participants’ lived experience of presence, immersion, and contemplative engagement, the primary aim of this review was to quantify effect sizes across standardized psychological and physiological outcomes to enable meta-analytic pooling. Qualitative data do not permit the calculation of standardized mean differences or the estimation of between-study heterogeneity that meta-analysis requires. This decision necessarily limits the review’s capacity to capture experiential dimensions of VRbMIs that may not be adequately represented by symptom-focused instruments. A complementary qualitative synthesis or mixed methods review remains an important direction for future research.

This study encompasses randomized controlled trials, nonrandomized controlled studies, and pre- and postintervention designs to reflect the methodological diversity of the emerging VRbMI literature. Qualitative-only studies, theoretical papers, reviews, protocols, conference abstracts, and dissertations without peer-reviewed publication were excluded. Nonimmersive digital interventions (eg, mobile or desktop mindfulness programs without VR), studies that did not involve a mindfulness-based practice, and studies lacking adequate reporting of outcomes were also excluded, and we limited inclusion to studies involving adult participants (aged 18 years and older), published in English.

### Search Strategy

A comprehensive search was conducted across 5 databases: PubMed, PsycINFO, Semantic Scholar, CORE, and Scopus—covering the period from January 2023 to April 2025. The Boolean framework used for all databases was:

- (“mindfulness-based intervention” OR “MBSR” OR “MBCT”)
- AND (“virtual reality” OR “VR” OR “immersive virtual reality”)
- AND (“stress” OR “anxiety” OR “depression” OR “HRV” OR “EEG” OR “cortisol”)

We manually searched reference lists of included studies and prior reviews (eg, Wieczorek et al [4]) to identify additional relevant publications and removed duplicates before screening.

### Screening and Study Selection

We screened titles and abstracts for relevance using predefined inclusion criteria and screened full text independently, applying the same criteria to confirm eligibility. A PRISMA 2020 flow diagram summarizes the number of records identified, screened, excluded, and included in the final synthesis. The author (SAB) represents the single screener, having performed the title, abstract, and full-text screening independently using predefined inclusion criteria. Artificial intelligence (AI)–assisted tools (Rayyan, SciSpace, and large language models) were used to support record organization and retrieval; however, we made all inclusion and exclusion decisions.

Of the 2943 records initially retrieved, 45 duplicates were removed, yielding 2898 unique titles and abstracts for screening. At the title and abstract stage, 2764 records were excluded. The most common reasons for exclusion were that the study did not use immersive VR technology, the study did not deliver a structured MBI, the publication type was ineligible (eg, review paper, editorial, theoretical piece, or protocol), or the study lacked sufficient data for synthesis. In cases where abstracts were unavailable, exclusion at this stage was limited to titles that clearly indicated misalignment with inclusion criteria. Of the 134 full-text papers assessed for eligibility, 99 were excluded using a structured tagging system within Rayyan. Exclusion reasons included mindfulness was peripheral or undefined within the intervention (excluded—score 1 peripheral), the study did not use immersive VR (excluded—not VR), the study lacked a structured MBI component (excluded—not MBI), or the full text was inaccessible due to paywall restrictions. A complete record of exclusion tags is available in the Open Science Framework (OSF) repository.

### Data Extraction

For each eligible study, data were extracted into a standardized spreadsheet, including authors, publication year, and country; study design and sample size; participant characteristics (age and population type); intervention characteristics (duration, frequency, headset type, environment description, and guidance mode); comparator type; psychological and physiological outcome measures; and pre- and postintervention means, SDs, and sample sizes. When statistics (eg, SDs) were missing, we estimated them from other reported values (eg, 2-tailed *t* statistics or η²) using formulas recommended in the Cochrane Handbook for Systematic Reviews of Interventions [15]. Additional variables extracted included participant characteristics, intervention features, and outcome measures. When data were missing or unclear, we applied conservative assumptions and standard estimation procedures and documented them during extraction. All extracted values were double-checked for accuracy. For each outcome domain, all reported results that permitted effect size calculation were extracted. When multiple measures or time points were reported within a study, we chose the most comparable pre-post or between-group results aligned with the primary analysis. In cases where studies reported results narratively as statistically significant without sufficient statistics for direct calculation, we applied a conservative estimate of *g*=0.5; for marginal or nonsignificant outcomes, we used *g*=0.30, following the precedent established by Borenstein et al [16]. Studies for which effect sizes could not be calculated or estimated were excluded from the quantitative synthesis and reported narratively.

### Data Preparation and Effect Size Computation

Following data extraction, all variables were organized with the explicit aim of computing standardized mean differences (Hedges *g*) for psychological outcomes across studies. Extracted data included pre- and postintervention means, SDs, and sample sizes for both within-subject and between-group designs, where available. When studies did not report sufficient statistical detail for direct calculation, consistent estimation procedures were applied to enable inclusion in the quantitative synthesis. We derived SDs from reported SEs or CIs using established formulas (SD=SE × √*n*; SD=[upper CI−lower CI]/[2×*t*_crit_]). For within-subject designs lacking a reported pre- and postcorrelation, a conservative estimate of *r*=0.50 was applied, consistent with recommendations for meta-analytic practice [15,17].

In cases where only test statistics or *P* values were reported, effect sizes were calculated using established transformation procedures [15,17]. When studies reported outcomes narratively without sufficient statistical detail, conservative estimates were assigned (*g*=0.50 for statistically significant effects; *g*=0.30 for marginal or nonsignificant findings), following precedent in prior meta-analytic work [4]. For outcome measures in which higher scores indicated greater symptom severity, effect sizes were reverse-coded so that positive values consistently reflected improvement. When multiple instruments assessed the same construct within a study, we selected the most used measure across the dataset to maintain comparability. All effect sizes were calculated as Hedges *g* with corresponding SEs and variances, which were subsequently used in random-effects meta-analytic models [14,18,19]. All pooled effects were evaluated using 2-tailed tests (α=.05). We used random-effects models to account for between-study variability.

### Risk-of-Bias Assessment

The methodological quality was evaluated using the MMAT 2018 [13]. Certainty of evidence was assessed qualitatively based on study quality (MMAT ratings), consistency of findings, and the extent of between-study heterogeneity. We rated each study across 5 domains: sampling, measurement, confounding, data completeness, and analysis. The ratings were summarized as low, moderate, or high risk of bias. When discrepancies arose during coding, we resolved them by verifying the source texts. Risk-of-bias assessments were conducted by a single reviewer (SAB) using the MMAT 2018 criteria. All ratings were performed independently, with AI-assisted tools used only to support identification of methodological details and not to determine final judgments.

### Effect-Size Calculation and Data Synthesis

We computed effect sizes for continuous outcomes as standardized mean differences (Hedges *g*) with corresponding SEs and 95% CIs. For within-subject designs, we assumed a conservative pre- and postcorrelation of *r*=0.50 when studies did not report this value. Random-effects models were used for all pooled analyses to account for expected between-study heterogeneity, estimating between-study variance with the DerSimonian-Laird method [14]. Although DerSimonian-Laird estimation is standard in small-sample meta-analyses, it may underestimate uncertainty under conditions of extreme heterogeneity; results should therefore be interpreted conservatively [14]. We included studies in the quantitative synthesis when sufficient statistical data were available to compute effect sizes. We retained studies that met the inclusion criteria but did not report adequate data for narrative synthesis and prepared data for synthesis by converting reported statistics into standardized mean differences (Hedges *g*). When summary statistics were incomplete, conservative estimation procedures were applied to derive effect sizes.

### Outcome Classification

We selected the 6 primary psychological outcome domains synthesized in this review—anxiety, depression, stress, negative affect, positive affect, and mindfulness—a priori based on 2 converging rationales. First, these domains were retained from Wieczorek et al [4] to ensure methodological continuity and enable direct comparison with the prior evidence base [4]. Second, they represent the outcome categories most consistently reported across the VRbMI literature, as identified during preliminary screening. Stress, anxiety, and depression reflect the dominant clinical targets of MBIs in Western psychological research [2,4]; positive and negative affect capture the broader affective profile of intervention response [4]; and mindfulness—assessed as both state and trait—directly indexes the contemplative construct central to all included interventions [1,2]. Domains such as sleep, cognition, and pain were reported by a subset of studies but were insufficiently represented across the sample to support meta-analytic pooling and were therefore retained for narrative description only. The distribution of outcome domains across the 35 included studies was as follows: anxiety (n=17, 49%), depression (n=17, 49%), stress (n=10, 29%), mindfulness (n=18, 51%), negative affect (n=4, 11%), and positive affect (n=4, 11%).

## Results

### Study Selection

**Figure 1.**
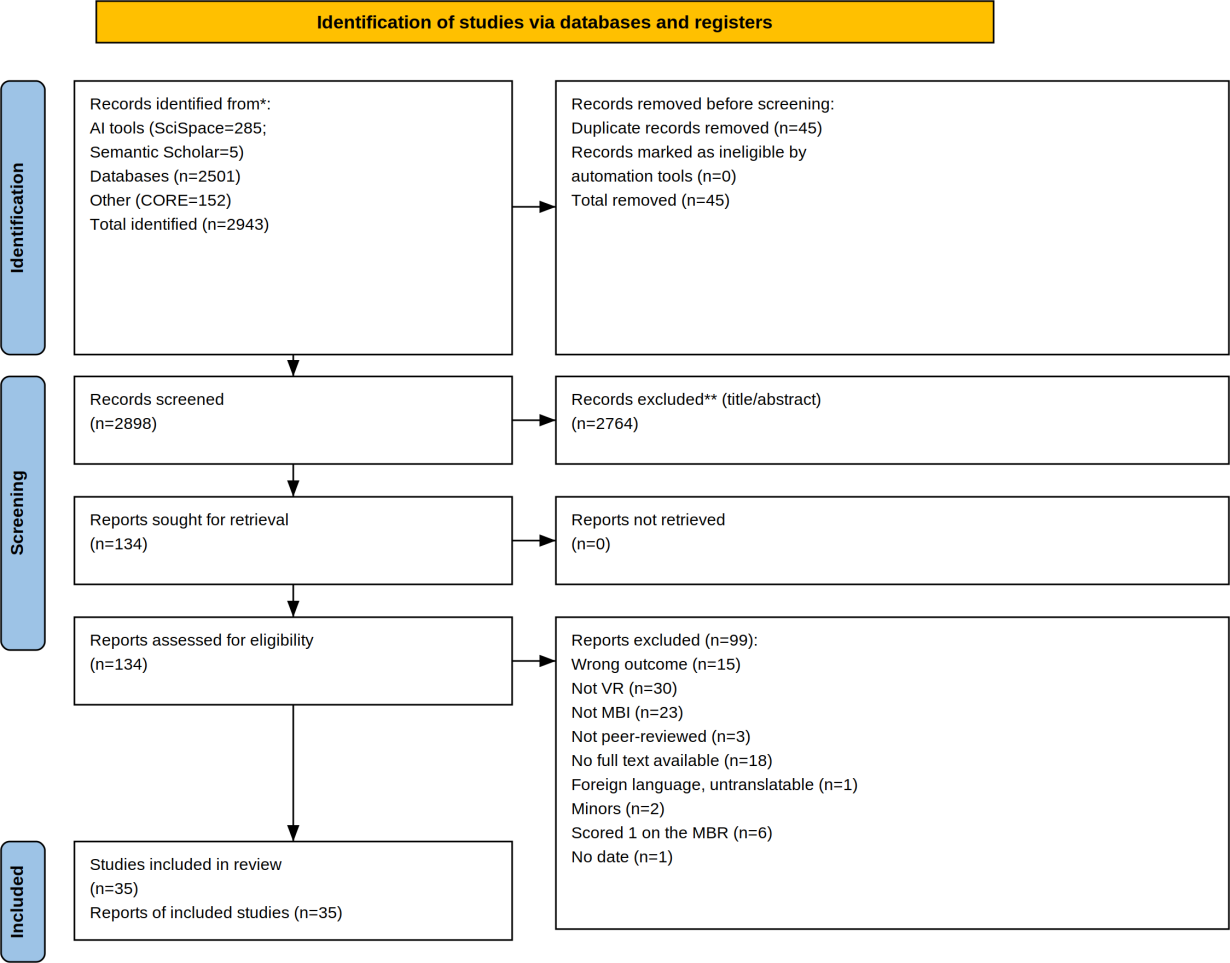
PRISMA 2020 flow diagram of study selection. The diagram depicts the number of records identified, screened, excluded, and retained in the final synthesis. *Records identified using AI-assisted tools (SciSpace, Semantic Scholar) and traditional databases. **Reasons for exclusion at the title/abstract stage were not individually categorized; the total reflects records not meeting VR-MBI criteria, empirical study requirements, or relevance thresholds. AI: artificial intelligence; MBI: mindfulness-based intervention; MBR: mindfulness centrality rubric; PRISMA: Preferred Reporting Items for Systematic Reviews and Meta-Analyses; VR: virtual reality.

Following the PRISMA 2020 reporting guidelines, study identification, screening, and inclusion were documented using a transparent flow diagram [12]. The search identified 2943 records from bibliographic databases and AI-assisted retrieval tools. After removing duplicates, we screened 2898 unique titles and abstracts and assessed 134 full-text papers for eligibility. Of these, 35 studies met all inclusion criteria and were included in the final synthesis. Figure 1 presents the PRISMA flow diagram summarizing the screening process and study selection. The completed PRISMA 2020 checklist is provided in Checklist 1.

### Study Characteristics

The 35 included studies encompassed a range of quantitative and mixed methods designs, including randomized controlled trials, quasi-experimental studies, and nonrandomized formats. The studies examined clinical, educational, and occupational populations, including individuals with depression, anxiety disorders, or psychosis [20,21], health care professionals experiencing burnout [22,23], patients with cancer [24,25], and university students engaged in mindfulness-based training [6,26,27]. McConnell et al [28] examined VR mindfulness for chronic low back pain in a 2-arm randomized trial, measuring disability and pain intensity across 12 sessions. Torres García et al [29] examined emotional discomfort in patients with breast cancer using the Emotional Discomfort Detection scale prior to first chemotherapy; while the VR group showed improvement trends, statistical reporting was insufficient for effect size computation, and the study was retained for narrative description only.

Geographically, the evidence base spanned North America, East Asia, and Europe, reflecting the growing international interest in VRbMIs. Studies were conducted in the United States [22,25,30-33], China [6,24,34], Taiwan [7], Finland [35], Spain [5,26,36], the United Kingdom [37], South Korea [21,38,39], and Turkey [40]. Supplementary visual summaries of study characteristics, including demographic figures and geographic distribution, are available in the OSF repository. Table 1 presents detailed characteristics of all included studies, including study design, population, intervention features, and outcome measures, in accordance with PRISMA 2020 reporting standards.

**Table 1.** Characteristics of included studies (N=35).

Study	Design	Values, n	Population	Intervention (dose)	Model	Guide	Comparator	Outcomes	Time	MMAT^a^	*g*
Ch et al (2023) [13]	Experiment	20	Remote workers	VR^b^ mindfulness (9 weeks)	360° video	Mixed	Control	Stress, creativity	Pre or post	Low-moderate	−0.57
Barton et al (2024) [35]	Mixed	58	Adults	VR mindfulness (1×6 minutes)	Mixed or multimodal	Guided	VR conditions	Mindfulness, fatigue, mood	Pre or post	Moderate	1.22
Blackmore et al (2024) [20]	Mixed	27	Clinical	VR mindfulness (1×15 minutes)	360° video	Guided	None	Anxiety, mood, mindfulness	Pre or post	Moderate	1.26
Cano et al (2024) [5]	Quasi-experiment	31	Post–COVID-19	VR mindfulness (16×60 minutes)	CAVE^c^	Guided	None	Cognition, attention	Pre or post	High	0.01
Cawley and Tejeiro (2024) [27]	Quasi-experiment	67	Students	VR mindfulness (1×10 minutes)	360° video	Guided	Audio, coloring	Stress, well-being	Pre or post	Low-moderate	0.34
Chen et al (2024) [41]	RCT^d^	76	Adults	VR mindfulness (8×18 minutes)	App-based VR	Guided	Control	Anxiety, depression	Pre or post+FU	Low	0.99
Ferrer Costa et al (2024) [23]	Pilot	83	Health care workers	VR mindfulness (8×13 minutes)	Immersive VR	Guided	None	Burnout, engagement	Pre or post	Low-moderate	−0.56
Fonseca et al (2024) [31]	RCT	26	Caregivers	VR mindfulness (1×6 minutes)	Immersive VR	Guided	Control	Anxiety	Pre or post	Moderate	−0.75
Franklin et al (2023) [25]	RCT	36	Patients with cancer	VR mindfulness (1×10 minutes)	Immersive VR	Guided	Control	Anxiety, depression	Pre or post	Low	NR^e^
Garland et al (2024) [32]	Pilot	34	SUD^f^	VR mindfulness (8×15 minutes)	App-based VR	Guided	None	Craving, mood	Pre or post	Low	0.53
Han et al (2023) [39]	Experiment	40	Students	VR meditation (1×10 minutes)	Immersive VR	Guided	Control	Anxiety	Pre or post	Moderate	N/A^g^
Hanna et al (2025) [37]	Quasi-experiment	32	Students	VR mindfulness (4‐8 weeks)	Immersive VR	Guided	No VR	Mindfulness	Pre or post	Moderate	N/A
Jimenez-Barragan et al (2025) [36]	RCT	70	Pregnant women	VR mindfulness (6 weeks, daily 14 minutes)	Immersive VR	Guided	Standard care	Pain, anxiety	Pre or post	Moderate	3.92
Kamada et al (2025) [42]	RCT	10	Patients undergoing surgery	VR mindfulness (NR^e^)	Immersive VR	Guided	Standard care	Pain, anxiety	Pre or post	Moderate	−1.49
Kim et al (2024) [38]	Experiment	8	Dementia	VR mindfulness (6 sessions)	Immersive VR	Guided	None	Anxiety, depression	Pre or post	Lo	N/A
Kim et al (2024) [43]	Experiment	60	Students	VR meditation (5×30 minutes)	360° video	Guided	Control	Sleep, HRV^h^	Pre or post	Low	1.28
Kumar et al (2024) [44]	RCT	40	Students	VR meditation (3×10 minutes)	Immersive VR	Guided	Control	EEG^i^, stress	Pre or post	Low-moderate	1.33
Lee et al (2023) [21]	RCT	64	Psychosis	VR mindfulness (8×30 minutes)	Mixed or multimodal	Guided	Control	Symptoms	Pre or post	Moderate	0.52
Liu et al (2025) [34]	RCT	90	Postpartum	VR mindfulness (8 weeks)	Immersive VR	Guided	Control	Anxiety, depression	Pre or post+FU^j^	Moderate	0.83
Mancini et al (2024) [45]	Experiment	21	Adults	VR mindfulness (NR)	Interactive	Guided	Audio	Mindfulness	Pre or post	Moderate	N/A
Mao et al (2024) [24]	Experiment	48	Patients with cancer	VR mindfulness (4×60 minutes)	360° video	Guided	None	Anxiety, fatigue	Pre or post+FU	Low-Moderate	1.93
McConnell et al (2024) [28]	RCT	52	Chronic pain	VR mindfulness (12	Mixed or multimodal	Guided	Standard care	Pain, disability	Pre or post	High	1.33
Modrego-Alarcón et al (2025) [26]	RCT	93	Students	VR mindfulness (6×7.5 minutes)	Immersive VR	Guided	Control	Mindfulness	Pre or post	Low	1.73
Murray et al (2024) [46]	Mixed	38	TBI^k^	VR mindfulness (4-minute sessions)	Immersive VR	Guided	None	Distress	Pre or post	Low	0.68
Ng et al (2024) [7]	Experiment	51	Students	VR breathing (4×6 minutes)	Immersive VR	Mixed	Control	EEG	Pre or post	Moderate	N/A
Olasz et al (2024) [8]	RCT	56	Students	VR mindfulness (2×20 minutes)	Immersive VR	Guided	Tablet	Anxiety, HR^l^	Pre or post	Moderate	1.07
Ong et al (2025) [47]	Mixed	35	Educators	VR mindfulness (1×10 minutes)	360° video	Guided	None	Mindfulness, UX^m^	Pre or post	Low	N/A
Poetar et al (2023) [48]	RCT	47	Adults	VR mindfulness (1×30 minutes)	App-based VR	Guided	Desktop	Mood	Pre or post	Moderate-high	0.58
Sexton-Radek et al (2024) [49]	Mixed	3	Students	VR mindfulness (7×15 minutes)	Immersive VR	Guided	None	Sleep	Pre or post	High	N/A
Sezer et al (2025) [40]	Experiment	54	Adults	VR mindfulness (1×20 minutes)	Immersive VR	Guided	Control	Anxiety, HRV	Pre or post	Moderate	0.50
Spitz et al (2024) [33]	Pilot	27	Dysphoria	VR mindfulness (2 weeks)	Immersive VR	Guided	None	Mood	Pre or post	Moderate	N/A
Torres García et al (2024) [29]	Quantitative (NR)	NR	Adults	VR mindfulness (NR)	Immersive VR	Guided	None	Emotional discomfort	Pre or post	High	N/A
Van Doren et al (2024) [30]	Mixed	32	Veterans	VR mindfulness (1×60 minutes)	360° video	Guided	None	Mindfulness	Pre or post	Moderate	−0.98
Williams et al (2024) [22]	Experiment	61	Health care workers	VR mindfulness (5‐15 minutes)	Immersive VR	Guided	None	Stress, engagement	Pre or post	Moderate	1.01
Zheng et al (2024) [6]	RCT	60	Students	VR mindfulness (2 weeks)	Immersive VR	Guided	Control	Anxiety, depression	Pre or post+FU	Low	1.81

a MMAT: Mixed Methods Appraisal Tool.

b VR: virtual reality.

c CAVE: room-scale CAVE system.

d RCT: randomized controlled trial.

e NR: not reported. Effect size could not be calculated due to the absence of a control or comparator group.

f SUD: substance use disorder.

g N/A: not available; effect size could not be calculated; study reported only *P* values and percentage change without group-level means and SDs necessary to compute Hedges *g*.

h HRV: heart rate variability.

i EEG: electroencephalography.

j FU: follow-up.

k TBI: traumatic brain injury.

l HR: heart rate.

m UX: user experience.

### Risk of Bias Assessment

Using the MMAT [13], we evaluated the methodological quality of all 35 included studies across 5 domains: sampling, measurement, confounding, data completeness, and analysis. Overall, most studies were rated as having a moderate risk of bias (n=22), reflecting partial adherence to quality criteria, commonly due to limited reporting of randomization procedures, incomplete follow-up data, or small sample sizes. In total, 11 studies met most or all MMAT criteria and were rated low risk. In contrast, we rated 3 studies as high risk due to inadequate control of confounding variables or incomplete reporting of outcomes. Among randomized controlled trials, methodological quality was generally strong, with clear descriptions of participant allocation and intervention fidelity. However, many trials did not report whether outcome assessors were blinded, and adherence monitoring was often limited. Nonrandomized designs exhibited a higher risk of bias in sampling and confounding domains, primarily due to convenience recruitment and the absence of active control conditions. Mixed methods studies demonstrated a moderate to high risk of bias, primarily due to the limited integration between the qualitative and quantitative components.

AI-assisted tools supported this appraisal by facilitating the identification of relevant methodological details within full-text papers; we reviewed all AI-assisted outputs before assigning final MMAT ratings. Table 2 summarizes the distribution of risk-of-bias ratings by study design.

**Table 2.** Risk of bias summary of study design.

Domain and score (low, moderate, or high)	Count, n
Quantitative randomized controlled trial	
High risk	1
Moderate risk	5
Low risk	8
Quantitative nonrandomized	
High risk	1
Moderate risk	13
Low risk	0
Quantitative nonrandomized or mixed methods	
High risk	1
Moderate risk	4
Low risk	2

### Quantitative Results

#### Overview

Following the risk-of-bias appraisal, quantitative outcomes were synthesized across the 35 included studies using standardized mean difference estimates. We conducted analyses using random-effects models to account for between-study variability and expressed all effect sizes as Hedges *g*, along with corresponding 95% CIs. The random-effects weighting was applied to minimize bias from unequal sample sizes and heterogeneous designs. When studies did not report complete statistics (eg, pre-post means, SDs, or sample sizes), we applied conservative estimation rules based on the direction and significance of reported effects, following Hedges [15] and guidelines for small-sample meta-analyses. All statistical transformations and pooling procedures were performed in JASP (version 0.19; University of Amsterdam) using the DerSimonian-Laird estimator for between-study variance [14], consistent with Hedges [15] framework and contemporary guidelines for small-sample meta-analyses [18].

The quantitative synthesis focused on 6 primary psychological outcome domains reported across multiple studies: anxiety, depression, stress, negative affect, positive affect, and mindfulness. Each outcome is summarized below, with pooled effect sizes, CIs, and heterogeneity statistics. Forest plots for all meta-analytic models, including study-level and pooled estimates, are available in the project’s OSF repository. The following sections present each outcome domain sequentially, beginning with anxiety, the most frequently reported psychological variable in the included studies. Studies contributing to each synthesis varied in design, sample characteristics, and risk of bias, with most rated low to moderate quality using MMAT criteria.

#### Anxiety

In total, 27 effect size estimates from 13 studies provided sufficient data for meta-analysis. A random-effects model indicated a small-to-moderate, nonsignificant pooled effect on anxiety (*g*=0.44, 95% CI –0.08 to 0.96; *t*_26_=1.74; *P*=.09). Between-study heterogeneity was substantial (*Q*_26_=381.60; *P*<.001; τ^2^=0.96), reflecting considerable variability in intervention design, population characteristics, and outcome measurement [15-17].

#### Depression

In total, 10 studies yielding 18 effect size estimates provided sufficient statistical data for meta-analysis. A random-effects model yielded a moderate pooled effect (*g*=0.62, 95% CI –0.13 to 1.36]; *t*_17_=1.75; *P*=.10); however, the CI crossed 0, indicating that the effect was not statistically significant. Between-study heterogeneity was substantial (*Q*_17_=236.77; *P*<.001; τ^2^=1.09) [15-17].

#### Stress

In total, 6 studies yielding 10 effect size estimates reported stress-related outcomes using validated self-report instruments, including the Perceived Stress Scale, the Depression, Anxiety and Stress Scale-21 stress subscale, or comparable measures [6,13,22,27,44,46]. Participant populations included university students, health care professionals, and community adults across both single-session and multiweek interventions. Individual study effect sizes varied across intervention formats. Some studies reported large reductions in perceived stress following immersive MBIs (eg, Kumar et al [44], *g*=1.33; Zheng et al [6], *g*=1.81), whereas others reported moderate or small effects following brief or lower-intensity interventions (eg, Murray and Shmidheiser [46], *g*=0.68; Cawley and Tejeiro [27], *g*=0.34). In occupational samples, effects were small or negligible [22]. A random-effects meta-analysis yielded a small-to-moderate pooled effect that did not reach statistical significance (*g*=0.45, 95% CI −0.08 to 0.99; *t*_9_=1.90; *P*=.09). Between-study heterogeneity was substantial (*Q*_9_=54.26; *P*<.001; τ^2^=0.35) [15-17].

#### Negative Affect

In total, 3 studies yielding 4 effect size estimates reported changes in negative affect using validated self-report instruments, including the Positive and Negative Affect Schedule (PANAS) and the Profile of Mood States [20,30,48]. Participant samples included university students, individuals enrolled in recovery programs, and nonclinical adult populations. Across studies, individual effect sizes indicated reductions in negative affect following MBIs delivered in VR and non-VR formats. Van Doren et al [30] reported a large pre- and postreduction following a brief immersive VR mindfulness session (*g*=–0.98), whereas Blackmore et al [20] observed a moderate reduction following a VR-supported mindfulness practice emphasizing curiosity and decentering (*g*=–0.60). Poetar et al [48] reported similar decreases in negative affect in both VR-based (*g*=–0.58) and desktop-based (*g*=–0.68) mindfulness conditions. A random-effects meta-analysis yielded a large and statistically significant pooled effect on negative affect (*g*=–0.67, 95% CI –0.92 to −0.42; *t*_3_=–8.71; *P*=.003). Between-study heterogeneity was negligible (*Q*_3_=0.93; *P*=.82; τ^2^=0.00) [15-17].

#### Positive Affect

In total, 3 studies yielding 4 effect size estimates assessed positive affect outcomes using the PANAS or comparable mood indices [20,30,48]. Participant samples included university students, clinical groups, and general adult populations. Individual study findings were variable. Van Doren et al [30] reported a moderate increase in positive affect following a single immersive mindfulness session (*g*=0.80). Poetar et al [48] reported a slight increase in the VR condition (*g*=0.27) and a slight decrease in the desktop-based comparison condition (*g*=–0.31). Blackmore et al [20] observed a slight, nonsignificant increase in positive affect (*g*=0.28). A random-effects meta-analysis yielded a small, pooled effect that did not reach statistical significance (*g*=0.24, 95% CI –0.45 to 0.92; *t*_3_=1.10; *P*=.35). Between-study heterogeneity was modest (*Q*_3_=6.58; *P*=.09; τ^2^=0.09) [15-17].

#### Mindfulness

In total, 18 studies assessed mindfulness-related outcomes using validated self-report instruments, including the State Mindfulness Scale, Five Facet Mindfulness Questionnaire, and Toronto Mindfulness Scale [20,26,27,30,35,37,45,47]. These measures captured both state and trait aspects of mindfulness following exposure to VRbMIs. Individual study findings varied across intervention formats and outcome measures. Cawley and Tejeiro [27] reported significant gains in state mindfulness following a brief VR session (*d*=0.69), whereas Blackmore et al [20] observed large pre- and posteffects for curiosity (*g*=1.26) and decentering (*g*=1.51). Modrego-Alarcón et al [26] reported smaller but measurable effects across multiple VR environments. Other studies reported modest changes following repeated exposure protocols.

In total, 2 studies yielding 5 effect size estimates using the State Mindfulness Scale provided sufficient data for quantitative synthesis. A random-effects meta-analysis yielded a large, statistically significant pooled effect on state mindfulness (*g*=1.00, 95% CI 0.32-1.68; *t*_4_=4.11; *P*=.02). Between-study heterogeneity was moderate (*Q*_4_=12.37; *P*=.02; τ^2^=0.21), reflecting variability in VR environment type and delivery format across conditions [15-17].

### Physiological Outcomes

Several studies included physiological measures alongside psychological outcomes; however, reporting was inconsistent and often incomplete. Standard indices included HR, HRV, electrodermal activity (or skin conductance level), EEG, and cortisol. Because most studies did not report sufficient statistics for quantitative pooling, physiological outcomes were summarized descriptively. Across studies, reported physiological changes generally corresponded with reductions in autonomic arousal following VR-based mindfulness exposure. Kim et al [38] reported increased HRV and reduced HR during guided VR meditation compared with a neutral VR control condition. Ng et al [7] observed decreased skin conductance levels and increased alpha EEG activity during mindfulness-based VR sessions. Williams et al [22] documented reductions in HR and respiration rate among health care professionals during immersive breath-awareness practice. Kamada et al [42] evaluated immersive VR for reducing intraoperative pain and anxiety in patients undergoing surgery in a single-center pilot randomized controlled trial conducted in Helsinki. Sexton-Radek et al [49] measured salivary melatonin, subjective calm, and sleep efficiency in a small mixed methods study, with participants reporting greater relaxation and modest improvements in sleep scores over 5 days; however, no statistical testing was conducted.

Only 5 studies provided analyzable physiological statistics, and baseline definitions varied substantially across designs. As a result, we did not conduct a formal meta-analysis of physiological outcomes. Heterogeneity in study design, small sample sizes, and variability in outcome reporting limited overall confidence in the evidence. Findings for stress-related outcomes supported greater confidence, while substantial heterogeneity across anxiety studies reduced confidence in those pooled estimates.

## Discussion

### Overview

This review synthesized evidence from 35 empirical studies examining VRbMIs to evaluate psychological outcomes and methodological quality. Across studies, VRbMIs were associated with favorable short-term psychological outcomes, most notably a large, significant reduction in negative affect and a large, significant increase in state mindfulness, alongside nonsignificant trends toward improvement in stress, anxiety, and depression. Pooled effect sizes ranged from small to moderate (*g*=0.24‐0.45) for affect, stress, anxiety, and depression outcomes, and large for negative affect (*g*=–0.67) and state mindfulness (*g*=1.00). Physiological outcomes, including changes in HR, HRV, and electroencephalographic markers, generally aligned with self-reported psychological findings; however, inconsistent and incomplete reporting precluded a quantitative synthesis. Taken together, the evidence suggests that VRbMIs can function as short-term supports for emotion regulation and attentional training. Although several pooled effects did not reach statistical significance, the overall direction and consistency of findings across studies, particularly for stress-related outcomes, support a cautious interpretation of short-term benefit. At the same time, these findings highlight methodological constraints that continue to shape how mindfulness is operationalized and evaluated within this emerging body of research.

### Psychological and Physiological Findings

Across diverse populations, including students, health care professionals, and clinical groups, VRbMIs showed directionally consistent improvements in stress, anxiety, and depressive symptoms, though pooled effects for these outcomes did not reach statistical significance consistent with the direction of prior meta-analytic findings [4]. Effects were generally larger in interventions that combined immersive environments with explicit mindfulness instruction or interactive guidance [6,20]. In contrast, outcomes related to positive affect and trait mindfulness were smaller and less consistent, suggesting that VRbMIs primarily support short-term emotional regulation rather than stable dispositional change. Most studies operationalized mindfulness using standardized psychological instruments such as the Depression, Anxiety and Stress Scale-21 (DASS-21), State-Trait Anxiety Inventory, and PANAS. While these measures facilitate cross-study comparability, they predominantly frame mindfulness in terms of symptom reduction and self-regulation. The inclusion rubric applied in this review excluded VR applications that focused solely on relaxation or entertainment, ensuring conceptual alignment with mindfulness-based practice.

Nevertheless, the resulting evidence base largely reflects a biomedical orientation that emphasizes stress management over broader contemplative dimensions. Of the 35 included studies, stress, anxiety, and depression were the 3 most frequently measured outcomes, while positive affect and mindfulness trait measures appeared in fewer than half of the studies, and no included study explicitly measured contemplative outcomes such as compassion, equanimity, insight, or nonattachment. This pattern mirrors broader critiques of secular mindfulness research, which has been characterized as prioritizing symptom reduction and clinical utility over the cultivation of wisdom, ethical development, and the liberative dimensions central to traditional Buddhist practice [3,50,51]. The dominance of distress-focused outcome measures reflects the influence of Western psychological frameworks, particularly cognitive-behavioral models on how mindfulness is operationalized and evaluated in digital health research.

Compared with Wieczorek et al [4], the present synthesis incorporates a broader and more methodologically diverse set of studies, including recent randomized trials and multisession interventions. While overall effect patterns remain broadly consistent, the inclusion of newer studies increases heterogeneity and reduces confidence in pooled estimates for several outcomes, particularly anxiety and mindfulness. These findings suggest that earlier estimates may have reflected smaller, more homogeneous samples.

### Methodological and AI Contributions

Despite rapid growth in VRbMI research between 2023 and 2025, methodological rigor across studies remained uneven. Common limitations included small sample sizes, brief intervention durations, limited follow-up, and reliance on single-group pre- and postdesigns. These features constrained statistical power and reduced confidence in causal inference. Even among randomized controlled trials, control conditions often failed to match VR interventions in terms of duration, engagement, or sensory load, complicating the interpretation of intervention-specific effects. Heterogeneity in outcome measures, intervention protocols, and reporting formats further limited comparability across studies. The methodological and epistemic dimensions of the AI-assisted review process used in this study are examined in depth in a companion paper currently under review.

### Conceptual Implications for Measurement and Design

Beyond procedural improvements, this review highlights a broader conceptual pattern in how mindfulness is represented within VR research. Nearly all included studies framed mindfulness as a culturally neutral attentional or stress-reduction technique, operationalized through symptom-focused instruments and evaluated against biomedical outcome criteria. While this approach supports cross-study comparability and clinical applicability, it narrows the construct of mindfulness to outcomes measurable within Western psychological paradigms. The epistemological and cultural dimensions of this narrowing, including how AI-assisted retrieval tools may reproduce dominant publication biases and how outcome measures embed assumptions about selfhood and well-being, are examined in depth in a companion paper currently under review. This analysis focuses on the methodological and empirical implications of these patterns for the design and synthesis of VRbMI research.

### Limitations and Future Directions

Several limitations constrain the interpretation of these findings. We did not conduct a sensitivity analysis due to the limited number of studies per outcome and substantial heterogeneity in study design and reporting, which constrained the feasibility of meaningful robustness testing. Many studies relied on small samples, brief follow-up periods, and incomplete statistical reporting, which reduced precision and limited the estimation of effect sizes. Substantial heterogeneity across interventions, outcome measures, and study designs further restricted comparability. Physiological outcomes showed potential value but were rarely reported with sufficient detail to support interpretation or synthesis. We did not conduct analyses to explore sources of heterogeneity (eg, subgroup analysis or meta-regression) due to the limited number of studies per outcome and variability in study designs. Further, we did not assess reporting bias (eg, publication bias) due to the small number of studies per outcome and heterogeneity in study designs.

At the review level, reliance on English-language publications likely introduced selection bias aligned with the dominant biomedical literature [52]. AI-assisted tools supported literature retrieval and screening. A complete methodological and epistemic analysis of this process appears in a separate paper currently under review and is therefore not reproduced here. Although the use of a structured inclusion rubric helped limit conceptual drift, future research should address both methodological and conceptual gaps. Larger trials with active control conditions and clearer reporting standards are necessary to clarify mechanisms and the durability of effects. For physiological measures in particular, a minimal reporting set specifying devices, sampling rates, preprocessing decisions, and measurement windows would enable cross-study comparison and longitudinal analysis. Beyond methodological rigor, future work should expand its conceptual scope by incorporating culturally grounded frameworks and qualitative or phenomenological methods capable of capturing the ethical, relational, and experiential dimensions of practice.

A further limitation concerns the sole-author design of this review. Although sole-authored systematic reviews are not uncommon in emerging fields, they introduce heightened risk of selection bias, confirmatory screening, and inconsistent application of inclusion criteria across a large study set. To mitigate these risks, we applied a structured, prespecified inclusion rubric documented in the PROSPERO registration and available in the OSF repository. We operationalized each criterion as a binary decision rule prior to screening and implemented a structured tagging system within Rayyan to document inclusion and exclusion decisions transparently at each stage. Each record was tagged with a specific reason code (eg, “not immersive VR,” “no mindfulness component,” and “insufficient data”) to ensure consistent and auditable decision-making throughout the screening process. Screenshots of the tagging workflow are available in the OSF repository. In cases where a study’s eligibility remained ambiguous, for example, when a VR application was described as “relaxation-based” but incorporated explicit breath-awareness or body-scan elements, we resolved uncertainty by returning to the primary source, applying the most conservative interpretation of the inclusion criterion, and documenting the decision rationale in the extraction log. Where ambiguity persisted after this process, we excluded the study. This approach prioritized consistency and transparency.

### Implications

These findings have implications for practice, research design, and theory. In applied contexts, VRbMIs provide a flexible format for supporting stress and emotion regulation in educational, clinical, and workplace settings. Their value lies less in novelty than in their capacity to scaffold attention and engagement for populations that may struggle with conventional practice formats. For researchers, the synthesis highlights the importance of standardized outcome reporting and transparent documentation of review processes. Without shared reporting conventions, especially for physiological measures, the field limits its ability to build cumulative knowledge. Transparency in both measurement and retrieval practices remains essential for interpretability and reproducibility. At a theoretical level, the review demonstrates that the tools used to study mindfulness also participate in defining it. Aligning intervention design and measurement with contemplative concerns such as attention, care, and interdependence may help balance methodological precision with conceptual integrity. Under these conditions, technological innovation can support the continuity rather than dilute the mindfulness practice.

### Conclusions

This synthesis of 35 empirical studies indicates that VRbMIs produce a large, statistically significant reduction in negative affect (*g*=–0.67; *P*=.003) and a large, statistically significant increase in state mindfulness (*g*=1.00; *P*=.02). Effects on depression (*g*=0.62; *P*=.10), stress (*g*=0.45; *P*=.09), and anxiety (*g*=0.44; *P*=.09) were small to moderate and did not reach statistical significance, though their direction was consistently positive. Effects on positive affect (*g*=0.24; *P*=.35) and trait mindfulness were small and less consistent, and physiological outcomes, while directionally aligned with self-reported findings, could not be formally synthesized due to incomplete reporting. Despite substantial heterogeneity across outcomes, the overall pattern of findings supports the use of VR-based mindfulness as a low-risk and accessible intervention across diverse populations. At the same time, the evidence base remains shaped by limited sample sizes, inconsistent physiological reporting, and a dominant biomedical framing that prioritizes symptom reduction over practice context. Future research that integrates rigorous trial design, transparent reporting practices, and culturally situated conceptual frameworks will better support both scientific credibility and theoretical coherence. Such integration may help ensure that VR-based mindfulness research advances not only technical effectiveness but also the broader purpose of mindfulness as a practice oriented toward awareness, responsibility, and human flourishing.

## Supplementary material

10.2196/90003 Multimedia Appendix 1 Mindfulness centrality rubric: inclusion criteria and screening procedures for virtual reality–based mindfulness interventions.

10.2196/90003 Checklist 1 PRISMA checklist.

## Abbreviations

AI: artificial intelligence
EEG: electroencephalography
HR: heart rate
HRV: heart rate variability
MBI: mindfulness-based intervention
MMAT: Mixed Methods Appraisal Tool
OSF: Open Science Framework
PANAS: Positive and Negative Affect Schedule
PRISMA: Preferred Reporting Items for Systematic Reviews and Meta-Analyses
VR: virtual reality
VRbMI: virtual reality–based mindfulness intervention
