# Supplementary material for: Efficacy of Virtual Reality–Based Mindfulness Interventions: Systematic Review and Meta-Analysis
Source: JMIR XR Spat Comput. 2026 Jun 24;3:e90003. doi: 10.2196/90003 (PMC13293560; doi:10.2196/90003)
Supplement: Multimedia Appendix 1 [file xr-v3-e90003-s001.pdf]

Appendix A: Mindfulness Centrality Rubric (MCR) for Inclusion in VR-Based Mindfulness Intervention Studies

To ensure consistency and transparency in study selection, this author created a Mindfulness Centrality Rubric and applied it during the full-text screening phase. This rubric evaluated whether the intervention under study implemented mindfulness as an explicit and structured component, grounded in formal contemplative practices. Only studies rated as 2 (Integrated)or 3 (Central) were included in the final synthesis. Studies rated as 1 (Peripheral) were excluded.

Protocol Mindfulness Centrality Rubric

Score	Description	Inclusion Status
3 – Central	Mindfulness is the core intervention; explicitly defined and grounded in MBSR, MBCT, or formal practice. Delivered through structured instruction (e.g., breath awareness, body scan, open monitoring). Outcomes include mindfulness or related constructs.	☑ Included
2- Integrated	Mindfulness is one of several active components (e.g., paired with psychoeducation, CT, or relaxation). Described as a meaningful part of the intervention or explicitly instructed. May or may not be measured directly.	☑ Included
1 – Peripheral	Mindfulness is mentioned but not defined, taught, or central to the experience. Often used interchangeably with “relaxation” or embedded in a passive environment without instruction.	✕ Excluded

Notes: 1). Rating was conducted during the full-text screening stage of the review. 2). Rubric scores were logged alongside other inclusion/exclusion decisions in the PRISMA-aligned tracking spreadsheet. 3.) Studies that did not include structured mindfulness practices (e.g., body scan, breath awareness, open monitoring, or loving-kindness meditation) were excluded.
